# Opto-Thermal Investigation of Additively Manufactured Steel Samples as a Function of the Hatch Distance

**DOI:** 10.3390/s22010046

**Published:** 2021-12-22

**Authors:** Dennis Höfflin, Maximilian Rosilius, Philipp Seitz, Andreas Schiffler, Jürgen Hartmann

**Affiliations:** 1 Institute of Digital Engineering, University of Applied Sciences Würzburg Schweinfurt, 97070 Würzburg, Germany; maximilian.rosilius@fhws.de (M.R.); philipp.seitz@fhws.de (P.S.); andreas.schiffler@fhws.de (A.S.); juergen.hartmann@fhws.de (J.H.); 2 Bavarian Center of Applied Energy Research e.V., 97074 Würzburg, Germany

**Keywords:** DMLS, additive manufacturing, laser flash method, thermal diffusivity

## Abstract

Nowadays, additive manufacturing processes are becoming more and more appealing due to their production-oriented design guidelines, especially with regard to topology optimisation and minimal downstream production depth in contrast to conventional technologies. However, a scientific path in the areas of quality assurance, material and microstructural properties, intrinsic thermal permeability and dependent stress parameters inhibits enthusiasm for the potential degrees of freedom of the direct metal laser melting process (DMLS). Especially in quality assurance, post-processing destructive measuring methods are still predominantly necessary in order to evaluate the components adequately. The overall objective of these investigations is to gain process knowledge make reliable in situ statements about component quality and material properties based on the process parameters used and emission values measured. The knowledge will then be used to develop non-destructive tools for the quality management of additively manufactured components. To assess the effectiveness of the research design in relation to the objectives for further investigations, this pre-study evaluates the dependencies between the process parameters, process emission during manufacturing and resulting thermal diffusivity and the relative density of samples fabricated by DMLS. Therefore, the approach deals with additively built metal samples made on an EOS M290 apparatus with varying hatch distances while simultaneously detecting the process emission. Afterwards, the relative density of the samples is determined optically, and thermal diffusivity is measured using the laser flash method. As a result of this pre-study, all interactions of the within factors are presented. The process variable hatch distance indicates a strong influence on the resulting material properties, as an increase in the hatch distance from 0.11 mm to 1 mm leads to a drop in relative density of 57.4%. The associated thermal diffusivity also reveals a sharp decrease from 5.3 mm^2^/s to 1.3 mm^2^/s with growing hatch distances. The variability of the material properties can also be observed in the measured process emissions. However, as various factors overlap in the thermal radiation signal, no clear assignment is possible within the scope of this work.

## 1. Introduction

The additive manufacturing technique, direct metal laser melting (DMLS), allows the layer-by-layer production of complex three-dimensional structures with almost bulk-like densities, using metal powder as raw material. This technology is increasingly applied to build material- and weight-efficient innovative parts without the use of additional tools, downstream process steps or clamping devices. Furthermore, functional integrations, such as casting moulds with included cooling channels, are possible.

As single powder layers are fused upon prior-built layers by laser heating, complex and time-dependent temperature profiles are introduced, which critically depend on process and material parameters [1]. The application of high laser intensities and scan velocities, resulting in exposure times in the range of milliseconds with extreme heating and cooling rates, cause unique microstructures and material properties [2].

However, these extreme process conditions can also have a negative impact on the manufacturing process. Until now, surveys indicate that process control and quality assurance are the most challenging technological barriers towards the successful application of additive manufactured parts in highly demanding or security relevant areas [3]. As a result, many researchers focus on developing methods for in situ monitoring systems [4]. For incorporating in situ non-destructive opto-thermal quality assurance, the understanding of the correlations between process parameters, resulting material properties and relating thermophysical properties is mandatory. In particular, the relative density and the thermal diffusivity are of high relevance. The aim of this preliminary study is to investigate the relationship between relative density, thermal diffusivity and emerging process emission during manufacturing by manipulating the hatch distance. Previous studies have dealt with different sub-aspects of this investigated field.

First, Krauss identified the hatch distance as a significant influencing factor regarding the density of additive manufactured components, as it influences the energy input as well as the distance between the heat-affected zones [5]. Zhou et al. investigated the effects of the hatch distance on microstructural features and mechanical properties of Ti-22Al-25Nb [6]. The results indicated that increasing the hatch distance leads to variations in relative density and microstructure, such as grain refinement and decreased texture intensity [6]. Finite element simulations have also shown that a larger hatch distance can lead to a higher temperature gradient and a smaller high-temperature range, which in turn is responsible for microstructural changes [6]. Ali et al. studied the effect of different parameter combinations on the porosity of selective laser melted Ti6Al4V [7]. The results showed a correlation between energy input and pore geometry. Here, low power and low exposure generated irregular shaped pores because of insufficient energy for melting, while excess heat input generated spherical pores [7].

Eucken described the effect of the pore geometry on the thermal conductivity in his contribution. He distinguished between spherical pores, elliptical and tubular pores as well as pores in the form of planar cracks, the latter having a stronger influence on thermal conductivity [8]. Further investigations on the thermal diffusivity determination of porous materials showed that the effective thermal diffusivity depends on the porosity of the samples, the form, size and distribution of pores as well as the thermal properties of the solid phase and the pores [9]. Bocchini et al. examined the thermal diffusivity of sintered steel samples with the laser flash method [10]. The presented thermal diffusivity model described a scheme of layered porosity oriented as a combination of serial and parallel configuration. The stated mathematical model based on their experimental results describe an exponential correlation between thermal diffusivity and density with three free fitting parameters *b*_1_, *b*_2_ and *b*_3_.

(1) $$\alpha =b_{1}+b_{2}\cdot e^{b_{3}\cdot \rho }$$

Bamberg et al. developed an in-process monitoring tool including an optical camera with high lateral resolution. Through long-term exposure and a correlation of the brightness of the different tracks with the energy input, small defects within the process were detected [11]. Volpp et al. observed melt pool temperature fields using a pyro-camera and an RGB camera to identify variations of process parameters in the melt pool dimensions of the temperature frames [12].

Based on the previous work, the research question of the present study was derived for the performed experimental method: is the approach of changing the hatch distance in combination with the recorded data of the emitted process radiation capable of predetermining the material properties mentioned above?

## 2. Materials and Methods

**Figure 1:**
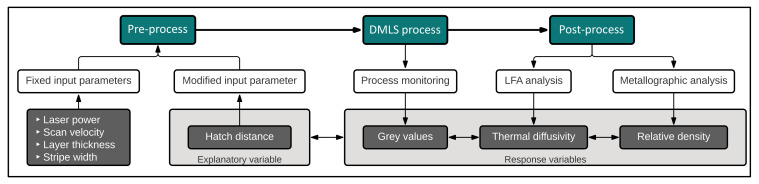
Flow chart of the experimental setup including the measured response variables.

The dedicated DoE of this pre-study based on a small sample size was chosen to initially verify the basic experimental design for feasibility and to pre-define constraints as well as regions of special interest. To investigate the dependency between relative density *ρ_rel_* and hatch distance Δ*h*, steel samples with different relative densities were produced by varying the hatch distance between two adjacent fusing lines with otherwise constant process parameters. The hatch distance of sample 4 (Δ*h* = 0.11 mm) was initially chosen because for the used setup, it is the characteristic value recommended and tested by the machine manufacturer EOS GmbH. Subsequently, the hatch distances were successively increased. Further, the occurring process emissions during manufacturing were recorded to relate the data to the measured material properties. After the manufacturing process, the samples were examined with regard to thermal diffusivity and relative density. Figure 1 shows a flow chart with the described process steps.

Table 1 shows the assignment of the hatch distances used with the respective associated volumetric energy densities. The volumetric energy density was derived from the process parameters laser power on path, scan velocity, hatch distance and layer height. 

**Figure 2:**
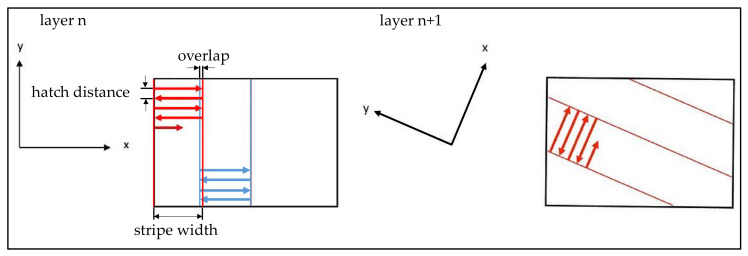
Schematic representation of the exposure strategy.

The DMLS-machine used was an EOS M290, including a 400 W fibre laser with a focal spot diameter of 100 µm. Further, an exposure strategy of rotating stripes with a continuous change of the scan direction between subsequent layers using a rotation angle of 67 degrees was applied. All samples were exposed alternately by dividing the surfaces into 10 mm wide exposure stripes with an overlap of 80 µm, illustrated in Figure 2.

**Figure 3:**
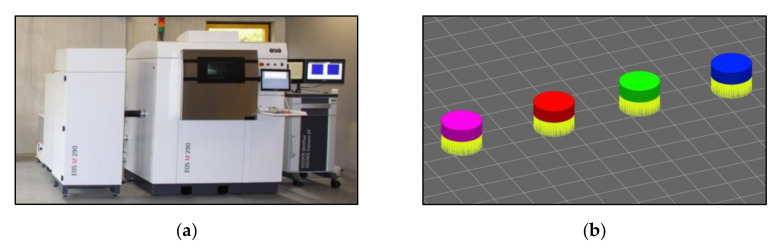
(**a**) EOS M290 including the EOSTATE Exposure OT System. (**b**) Representation of the samples. Section from the data preparation. Left to right: Samples 1–4.

Additional process parameters were a scan velocity of 960 mm/s, a laser power of 285 W and a thickness of the powder layers of 40 µm. The used material was EOS MaragingSteel MS1 with a grain size ≤63 µm, which corresponds to the material composition according to the European classification 1.2709. The exact composition is given in Table 2. A cylindrical geometry of the samples was used with a diameter of 12.7 mm and a height of 4 mm. Figure 3 shows the DMLS machine EOS M290 used for manufacturing and samples as an image section from the data preparation. The different colours represent the different parameter sets. Further, a sample made of conventional 1.2709 material (melted under vacuum, forged and shaped by CNC milling) served as reference for the thermal diffusivity data.

The relative densities of the additively fabricated samples were measured optically. Therefore, the samples were cut perpendicular to the built direction, embedded in epoxy, grinded and polished. The microscopic investigation was performed using a microscope (Olympus AX70). To calculate the relative density of the additively manufactured samples, the ratio of the pore area to the dense area of the material was used. For the conventional sample, the relative density was determined using Archimedes’ method, i.e., measuring the weight in air and water.

The process monitoring was performed with the EOSTATE Exposure OT [14], a system for quality assurance and control during fabrication of additive manufactured parts. It consists of a sCMOS camera, which measures the emitted process radiance during exposure with 100 fps at a centre wavelength of 900 nm and a narrow bandwidth of 25 nm. The values of the process emission are subsequently integrated pixel-by-pixel over the exposure time for each layer. Correlating the brightness with the multiplication of spectral radiance and time, the resulting grey values can be used to characterise the process [10].

Changing the hatch distances not only changes the volumetric energy density of a single layer but also the area of exposure and the working time of the laser as well as the integration time of the OT pictures. Therefore, the averaged grey scale of a single layer was correlated to the working time *t_Sample,n_* of the laser on each sample layer *n*. Here, a bisection of the hatch distance led to a duplication of the laser working time. Thus, the increased average grey values of the individual layers due to the increasing exposure times were taken into account in order to draw conclusions about appearing temperature differences. The exposure time of the laser *t*_1*,n*_ of Sample 1 on a layer *n* with a hatch distance Δ*y*_1_ = 1 mm was used as a reference value.

The opto-thermal evaluation was carried out with a laser flash apparatus (NETZSCH LFA 427), which is commonly used to measure the thermal diffusivity of a variety of different materials [15]. In this method, a short laser pulse at the front side heats a plane-parallel sample and the resulting temperature rise at the sample backside is measured. The higher the thermal diffusivity of the sample, the faster the temperature rise at the backside. For the one-dimensional, adiabatical case, the thermal diffusivity *α* can be calculated by [16]

(2) $$\alpha =0.1388\frac{d^{2}}{t_{0.5}}$$

Here, *d* is the sample thickness and *t*_0.5_ is the time needed for reaching half of the maximum temperature rise. In the steps of Δ*T* = 50 °C, the thermal diffusivity of four additively built samples with different relative densities plus one bulk reference sample were measured. The observed temperature range was between 50 °C and 600 °C. The nominal density of the full dense material was taken as *p* = 8.1 g/cm^3^ and the specific heat capacity as *c_p_* = 450 J/kgK [13].

## 3. Results

### 3.1. Presenting Data

#### 3.1.1. Overall

Table 3 shows the results of the data obtained from the measurements. The thermal diffusivity at 300 °C is presented exemplarily, as it is the median of the temperature range of the measurement. The full data are given afterwards.

#### 3.1.2. Relative Density

**Figure 4:**
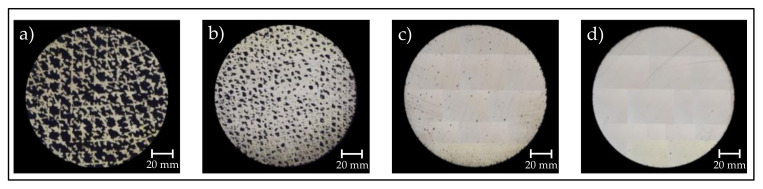
Micrographs of the additively fabricated samples in order to distinguish the relative densities by measuring the pore area in relation to the dense material area. (**a**) Sample 1, *ρ_rel_* = 42.3%. (**b**) Sample 2, *ρ_rel_* = 82.2%. (**c**) Sample 3, *ρ_rel_* = 99.1%. (**d**) Sample 4, *ρ_rel_* = 99.7%.

Due to the different hatch distances and the resulting different volumetric energy densities, the fabricated samples have different relative densities. The micrographs used for the measurement of the relative density, including the results for Samples 1–4, are shown in Figure 4. The Archimedes measurement of the conventional sample resulted in a density of 8.1 g/cm^3^ and was taken as a relative density of 100%.

#### 3.1.3. Thermal Diffusivity

**Figure 5:**
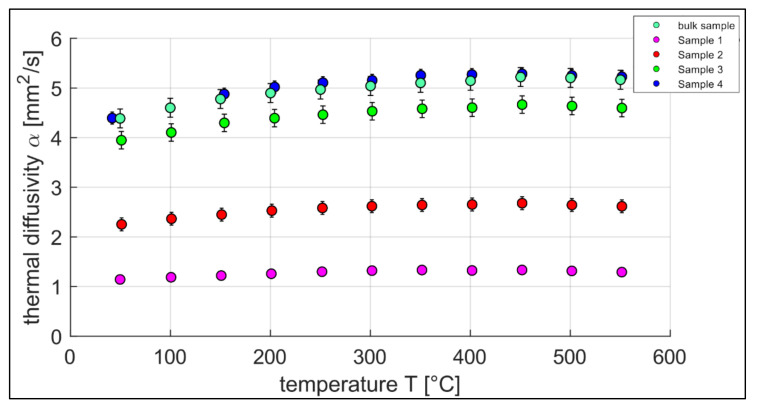
Thermal diffusivity of additive manufactured samples and the conventional bulk sample made out of MaragingSteel MS1 resp. 1.2709 over the temperature, measurement accuracy = 3%.

Figure 5 shows the thermal diffusivities *α* of the additive and the conventional manufactured samples as a function of temperature. The resulting curves support the statement of the literature [9], saying the thermal diffusivity of porous materials increases with decreasing porosity. Based on that statement, the maximum value is the thermal diffusivity of the completely dense sample. Here, Sample 4 and the bulk sample show the highest thermal diffusivities over the measured temperature range with slightly higher values within the combined uncertainty for the additively manufactured Sample 4. The reason for the varying thermal diffusivities despite the same basic material lies in the different microstructure resulting from the different manufacturing processes (Sample 4—Direct metal laser melting; conventional sample—Vacuum melting, forging and CNC-milling), which has a major impact on the thermal diffusivity of steel [17].

#### 3.1.4. Process Emission

**Figure 6:**
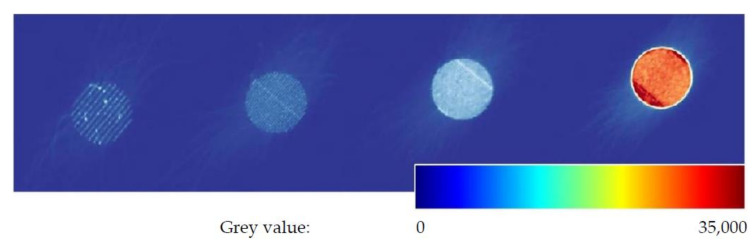
Integrated picture of the monitoring system for a single layer. From left to right: Sample 1 to 4.

**Figure 7:**
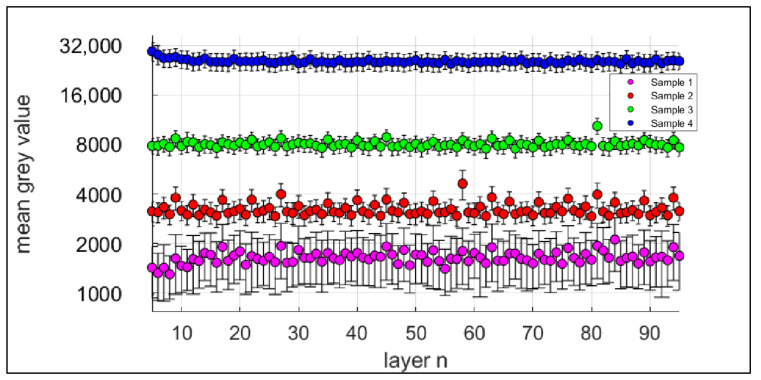
Mean grey values of the OT-process monitoring system of the layers 5–95.

Since one of the objectives of this study is to correlate the process emission during manufacturing with the resulting material characteristics, Figure 6 shows an exemplary OT image of a single layer. Furthermore, the measured mean grey values of all samples for each component layer are presented in Figure 7.

Planck’s law of thermal radiation describes the correlation of spectral radiance of thermal radiation, i.e., thermal power, per solid angle, area and wavelength interval of a black body and its temperature as a function of wavelength. According to that, the differences in pixel brightness can be correlated to temperature differences during the fabrication process. Due to the rapidly changing state of aggregation and the changing emissivities in the meltpool as well as the high spatial and temporal temperature gradients, it is not straightforward to determine absolute temperatures. Further, caused by the small distance between adjacent fuse lines and the resulting overlap of the heating zones during the integration time, some pixels observe multiple staggered heating and cooling phases. Therefore, it is not possible to correlate the resulting pixel grey values to a single temperature at a defined time during fabrication of a single layer. However, the following statement still holds: the higher the temperature during exposure, the higher the radiance emitted from the meltpool and the surrounding material that is detected by the thermal camera. Figure 7 shows the averaged mean grey values for layers 5–95 of the four additively fabricated samples. As expected, the measured grey values rise with increasing volumetric energy density.

As previously described, Table 4 shows the normalised mean grey values in relation to the exposure time *t*_1*,n*_ of the first sample. It can be observed that the range of the normalised mean grey values between the individual samples becomes significantly narrower with the lowest value at Sample 2.

### 3.2. Visualisation and Fitting Models

**Figure 8:**
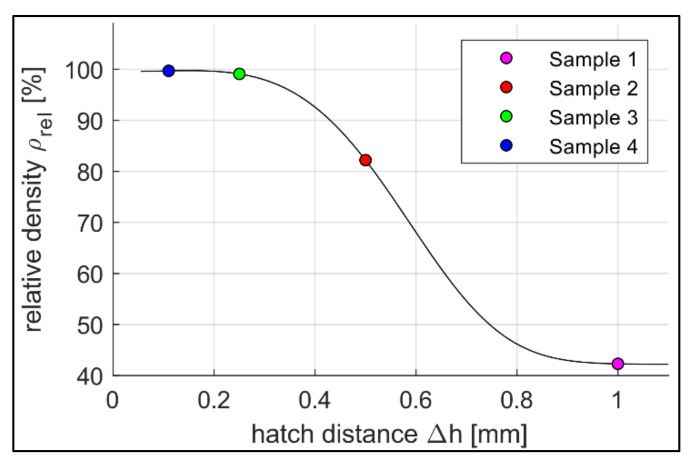
Distribution of the relative density *ρ_rel_* over the hatch distance Δ*h* for Samples 1–4, fitting curve equation: $f_{3}\left(x\right)=e^{\left(-c_{1}\cdot x^{4}+c_{2}\cdot x^{2}+c_{3}\right)}+c_{4}$ (3); c_1_ = 7.0303, c_2_ = 0.3162, c_3_ = 4.0485, c_4_ = 42.2304; r = 1.8778 × 10^−21^; R^2^ = 100.

**Figure 9:**
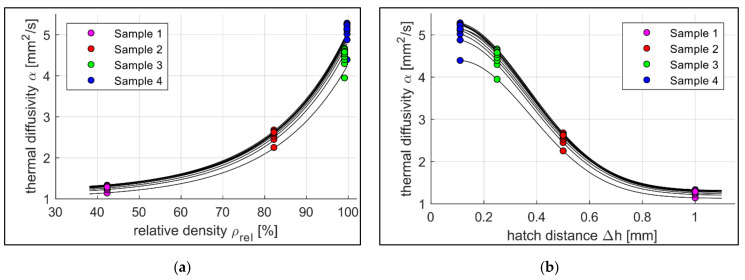
(**a**) Diffusivity over relative density of the samples 1-4. Measured temperature range (bottom up) 50–600 °C, step size of 50 °C, set of fitting curves Equation: $f_{4}\left(x\right)=e^{\left(c_{1}\cdot x+c_{2}\right)}+c_{3}$ (4); c_1,mean_ = 0.0554, c_2,mean_ = −4.2109, c_3,mean_ = 1.1370; r_mean_ = 0.1207 ± 0.0517; R^2^ = 98.69 ± 0.4319 (**b**) Thermal diffusivity *α* over hatch distance Δ*h* for different temperatures of Samples 1–4. Measured temperature range (bottom up) 50–600 °C, step size of 50 °C, set of fitting curves Equation: $f_{5}\left(x\right)=e^{\left(-c_{1}\cdot x^{2}+c_{2}\cdot x+c_{3}\right)}+c_{4}$ (5); c_1,mean_ = 5.56, c_2,mean_ = 0.89, c_3,mean_ = 1.20, c_4,mean_ = 1.16; r_mean_ = 0.6400 × 10^−16^ ± 4.2455 × 10^−16^; R^2^ = 100 ± 8.9877 × 10^−15^.

**Figure 10:**
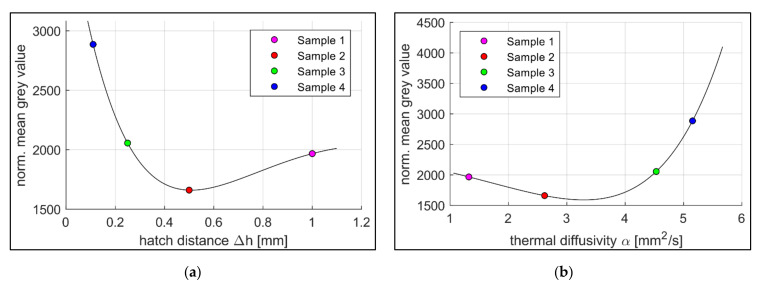
Normalised mean grey values of the samples 1–4 (**a**) over hatch distance Δ*h*, fitting curve Equation: $f\left(x\right)=(c_{1}\;x^{2}\;c_{2}\;x+c_{3})\cdot e^{\left(-c_{4}\cdot x\right)}$ (6); c_1_ = 13461.87, c_2_ = 7398.36, c_3_ = 4108.68, c_4_ = 1.64; r = 4.12 × 10^−20^; R^2^ = 100, effect regions according to (**b**) over the thermal diffusivity *α*, fitting curve according to Equation: $f\left(x\right)=(c_{1}\cdot x^{2}-c_{2}\cdot x+c_{3})\cdot e^{\left(c_{4}\cdot x\right)}$ (7); c_1_ = 98.7, c_2_ = 826.9, c_3_ = 2227.6, c_4_ = 0.3; r = 4.47 × 10^−23^; R^2^ = 100.

The following Figure 8, Figure 9 and Figure 10 show the graphical representations of the measurement results. For reasons of clarity, empirical fitting curves were determined and additionally drawn for the individual combinations. These curves do not describe a mathematical model based on physical conditions. They are merely intended to provide a first estimation of the relationships between the individual parameters. In order to further elaborate the various correlations, different parameter combinations are plotted with respect to each other. Figure 8 shows the relative density *ρ_rel_* over the hatch distance Δ*h*. 

In Figure 9a, the thermal diffusivity *α* is plotted with respect to the relative density *ρ_rel_* of Samples 1–4 for every temperature step over the measured temperature range between 50 °C and 600 °C. Further, a set of fitting curves, based on the model of Bocchini is presented [9]. Figure 9b shows the relation of the thermal diffusivity *α* over the hatch distance Δ*h*, including another set of fitting curves. Similar to Figure 9a, the measured values of the four samples are displayed in temperature steps of 50 degrees from 50 °C to 600 °C. As the thermal diffusivity of the samples rise with increasing measurement temperatures (cf. Figure 4), the lowest measurement temperature belongs to the lowest curve and the highest temperature to the upper curve. These findings match the results of Jarfors et al. measuring the temperature dependency of the thermal diffusivity of Maraging steel (1.2709) [18].

Figure 10 illustrates the normalised mean grey values recorded during the production of Samples 1–4 (a) over the associated hatch distances Δ*h* and (b) over the thermal diffusivity *α* at 300 °C. 

### 3.3. Interpretation

#### 3.3.1. Statistics

In the following, the results are statistically evaluated. As a premise of this study, a preliminary approximation of a correlation analysis confirms a non-linear correlation (Spearman’s *ρ* = −1; *p* = 0.083) of the manipulation of the relative density by the hatch distance. In Figure 8, the plausibility of the constraint for the hatch distance towards 0 is also given. As an additional physical basis of the experimental design, the linear relationship between the volumetric energy density and the mean grey value was also confirmed to be highly significant (Pearson’s *ρ* = 0.998; *p* = 0.002). The postulated correlation of thermal diffusivity and hatch distance (Spearman’s *ρ* = −1; *p* = 0.083) was proven within the samples to a first estimation. In addition, the correlation between thermal diffusivity and relative density within the samples (Spearman’s *ρ* = 1; *p* = 0.083) was obtained (cf. Figure 9b). Nevertheless, further tests with larger sample sizes are recommended for a more comprehensive investigation.

#### 3.3.2. Effect Interpretation

Figure 8 shows the correlation between the hatch distance and relative density, where the relative density decreases exponentially with rising hatch distance. Increasing the distance between two adjacent fusing lines results in incompletely melted metal powder as well as a rising number of defects and occlusions. This causes higher porosity, which is equivalent to a reduction in the relative density. For very small hatch distances towards 0 mm, the relative density is expected to reduce again, as too much energy input overheats the process. This in turn leads to increasing defects, such as keyhole pores in the material [6].

**Figure 11:**
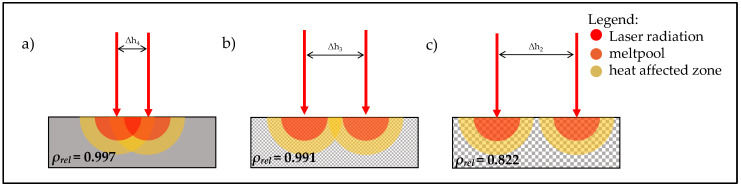
Schematic illustration of the superposition of two time shifted heating processes. Δh_4_ < Δh_3_ < Δh_2_. (**a**) superposition of adjacent melting zones (**b**) superposition of adjacent heat affected zones (**c**) no superposition of adjacent heat affected zones.

In Figure 9a, the values of the thermal diffusivity of Samples 1–4 are plotted with respect to the relative density at different measurement temperatures, including a set of fitting curves. The data presented suggest an exponential relationship between the variables. This agrees with the findings of Beiss [19] and Bocchini [10], whereas the thermal diffusivity increases exponentially with rising relative density. The evaluation of the results of Figure 10a shows that the normalised mean grey values and the correlating process temperatures of the first two samples differ significantly. Sample 1 shows higher values despite lower volumetric energy input. The reason suspected is a material-dependent effect. The findings of Figure 9b show that thermal diffusivity decreases with increasing hatch distance. As a result, the laser-induced heat dissipates more slowly into the material, causing the surface to heat up more. This increases the normalised mean grey values. If only this effect was taken into account, a further drop in grey values with decreasing hatch distances and, therefore, increasing thermal diffusivities would be expected. However, the results show a steep rise in the normalised mean grey values for smaller hatch distances. The presumed cause here is an overlay effect. Samples 1 and 2 have large hatch distances and, therefore, large distances between two adjacent fusion lines (Δ*h_1_* = 1 mm for Sample 1 and Δ*h_2_* = 0.5 mm for Sample 2). The fusion lines with a meltpool width of approximately 180 µm to 200 µm and their respective heating zones do not interact. That allows the heat to dissipate into the solid material without further heating the component due to superimposition (cf. Figure 11c). At hatch distances around Δ*h_3_* = 0.25 mm (Sample 3) the heating zones start to overlap as the fusion lines approach (cf. Figure 11b). This leads to multiple time shifted heating and cooling phases that sections of the material receive. As the monitoring system integrates the emission for every pixel over the complete layer, the normalised mean grey values rise. At a hatch distance of Δ*h_4_* = 0.11 mm (Sample 4), the neighbouring fusion lines start to touch each other, which maximises the overlapping of the heat-affected zones (cf. Figure 11a). In addition, the high volumetrically introduced energy density in combination with the short scan vectors causes the material to be unable to dissipate the heat into the material in time despite the high relative density. This leads to local hot spots with increased temperatures and higher normalised mean grey values.

Figure 10b also shows the two effects described above, which mainly determines the course of the normalised mean grey values (here, in relation to the thermal diffusivity). While at lower thermal diffusivities the material dependent effect predominates, since the temperature-affected zones do not overlap, the overlay effect becomes more and more decisive with rising thermal diffusivities.

## 4. Discussion

In this study, samples with different properties were produced by varying the hatch distance. The relationship between hatch distance and relative density follows the results given by Zhou et al. [6] and Huang [20]. According to them, an increase in the hatch distance away from the optimum value is always accompanied by an increase in porosity. The changed thermal diffusivity was also investigated as part of this work. The highest values were found at the same hatch distances as the samples with the highest relative density. These results are consistent with the statements in the literature according to which the thermal diffusivity as well as the thermal conductivity of a sample is strongly dependent on its porosity [8,10]. A further amplification effect towards the sharp rise in the thermal diffusivity in the region of small hatch distances can be explained by the dependence of thermal diffusivity on pore shape [9]. Spherical pores affect the thermal diffusivity of materials with the same porosity less than irregularly shaped pores [8]. The predominant presence of spherical pores at the small hatch distance are induced within the findings of Ali [7] and Huang [20]. According to their work, high process energies, among others, caused by small hatch distances lead to spherical pores due to collapsing keyholes. On the contrary, large hatch distances tend to lead to a lack of fusion and, thus, to larger irregularly shaped pores. 

Finally, the results within the framework of this study confirm a similarity of the given mathematical correlations between porosity and thermal diffusivity of Bocchini [10]. The reason for the discrepancies in the coefficients is found in the different material and manufacturing process. Bocchini used a sintering process for production, which means that deviations in the pore shape cannot be excluded. However, the sample size is too small for a more detailed statement.

Moreover, the process emission during the manufacturing process was recorded and compared to the process parameters used as well as the material properties obtained. The measurement data show a clear correlation between hatch distances and normalised grey values (cf. Figure 10a). Especially in the range around the optimum value for the hatch distance (Sample 4, Δ*h* = 0.11 mm), the measured grey values change to a great extent. The thermal diffusivity shows a comparably strong dependence with the largest changes of the normalised grey values at the highest values of thermal diffusivity (cf. Figure 10b). Bamberg et al. used a comparable measuring system to the one presented here, but the focus was on detecting local anomalies within the individual layers [11]. That way, he succeeded in drawing conclusions about material defects through altered grey values. However, the approach of the study carried out here initially refers to complete layers and the comparison of different components with each other. The local changes in the measured grey values are nevertheless included in the total grey value and, thus, contribute to the characterisation of the components among each other.

## 5. Conclusions

In this pre-study, EOS MaragingSteel MS1 was fabricated by DMLS using different hatch distances. At the same time, the process emissions were measured, and subsequently the material parameters’ relative density and thermal diffusivity were determined. Based on the results obtained, the following conclusions can be drawn:

- Relative density and thermal diffusivity decrease with increasing hatch distance.
- Thermal diffusivity increases with increasing relative density.
- The correlations of the detected process emissions with respect to the hatch distance as well as description approaches of superimposed interaction effects of the heat transfer and component properties are presented. The normalised mean grey values follow a bathtub curve-like course, which is created by overlapping effects.

Overall, the results confirm the previous findings from the literature and indicate the suitability of the experimental design for the previously formulated objectives. The resulting correlations and their causality need to be investigated more closely with a sufficient sample size in a future study. In this context, the boundary conditions in the border areas as well as in the region around the point of interest should be examined in more detail. Further variables, such as the dependency of the pore structure and dynamic hysteresis behaviour of the above-mentioned distributions, should also be taken into account. A further application-related benefit of these research results is a possible anticipation of desired target values (thermal diffusivity and relative density) through design and manufacturing derivations.

## Figures and Tables

**Table 1:** Sample notations, hatch distances and resulting volumetric energy densities.

Sample Designation	Hatch Distance Δ*h* [mm]	Volumetric Energy Density [J/mm^3^]
Sample 1	1	7.42
Sample 2	0.5	14.84
Sample 3	0.25	26.69
Sample 4	0.11	67.47

**Table 2:** Chemical composition of EOS MaragingSteel MS1 [13].

Alloying Element	Fe	Ni	Co	Mo	Ti	Al	Cr	C	Mn, Si	P, S
wt%	rest	17–19	8.5–9.5	4.5–5.2	0.6–0.8	0.05–0.15	≤0.5	≤0.03	≤0.1	≤0.01

**Table 3:** Overview of the acquired data. Sample designation, hatch distance Δ*h*, volumetric energy density, resulting relative density $\rho_{rel}$, averaged mean grey value over all layers and thermal diffusivity *α* at 300 °C.

Sample Designation	Hatch Distance Δ*h* [mm]	Volumetric Energy Density [J/mm^3^]	Relative Density *ρ_rel_* [%]	Mean Grey Value [—]	Thermal Diffusivity *α* at 300 °C [mm^2^/s]
Sample 1	1	7.42	42.3	1967	1.321
Sample 2	0.5	14.84	82.2	3317	2.619
Sample 3	0.25	26.69	99.1	8219	4.532
Sample 4	0.11	67.47	99.7	26,134	5.256
Conv. sample	-	-	100	-	5.041

**Table 4:** Correlation of relative densities to the mean grey values normalised to the exposure time t_1_ of the fabrication laser and the thermal diffusivity at 300 °C.

Sample Designation	Relative Density *ρ_rel_* [%]	Normalised Mean Grey Value	Thermal Diffusivity *α* [mm^2^/s] at 300 °C
Sample 1	42.3	1967	1.321
Sample 2	82.2	1660	2.619
Sample 3	99.1	2056	4.532
Sample 4	99.7	2886	5.256

## Data Availability

The data presented in this study are available on request from the corresponding author.

## Nomenclature

Symbol	Property	Units
*α*	Thermal diffusivity	$\frac{m^{2}}{s}$
Δ*h*	Hatch distance	mm
*d*	Sample thickness	mm
t^0,5^	Time to half maximum	s
t_Sample,n_	Laser working time	s
Δ*T*	Temperature difference	K
*c_p_*	Specific heat capacity	$\frac{J}{\mathrm{kg}\cdot K}$
*ρ*	Specific density	$\frac{g}{cm^{3}}$
*ρ_rel_*	Relative density	%
*r*	Residuum	-
*R* ^2^	Coefficient of determination	%
